# A Novel Case of Symptomatic BK Viraemia in a Patient Undergoing Treatment for Hodgkin Lymphoma

**DOI:** 10.1155/2014/909516

**Published:** 2014-06-24

**Authors:** Jacinta Perram, Jane Estell

**Affiliations:** ^1^St Vincent's Hospital Sydney, 390 Victoria Street, Darlinghurst, NSW 2010, Australia; ^2^Department of Haematology, Concord Hospital, Hospital Road, Concord, NSW 2137, Australia

## Abstract

Symptomatic BK viral infection in the immunocompromised host is well described, most commonly seen in renal transplant recipients, bone marrow transplant recipients, and HIV positive patients. The present case describes a novel clinical scenario of symptomatic urological BK virus infection in a patient receiving treatment for Hodgkin lymphoma. This case highlights the importance of casting a wide diagnostic net for adverse events encountered with novel therapeutic agents or regimens.

## 1. Case Presentation

B-K, a 38-year-old male, presented with symptoms of severe bladder cystitis over a 2-week period, during treatment for nodular sclerosing Hodgkin lymphoma stage IVB. At the time of presentation, he had completed four cycles of escalated BEACOPP and was receiving the second of four planned standard BEACOPP cycles.

The escalated BEACOPP regimen consists of doxorubicin 35 mg/m^2^ and cyclophosphamide 1250 mg/m^2^ on day 1, etoposide 200 mg/m^2^ on days 1 to 3, procarbazine 100 mg/m^2^ on days 1 to 7, vincristine 1.4 mg/m^2^ and bleomycin 10 0000 IU/m^2^ on day 8, and prednisone 40 mg/m^2^ on days 1 to 14. Doses of cyclophosphamide, doxorubicin, and etoposide are decreased in the standard regimen. Assessments after four cycles of escalated BEACOPP revealed that the patient was both PET and CT negative for Hodgkin lymphoma.

Treatment cycles 1–4 were well tolerated with expected grade 3 or grade 4 hematological toxicities and no infective complications. Of note lymphopenia of 3–12-day duration occurred, with a nadir of 0.1 × 10^9^/L each cycle. Bleomycin was omitted in cycle 6 due to pulmonary toxicity. Two admissions were required for upper respiratory tract infection during cycle 5: the first was treated with piperacillin/tazobactam, gentamicin, amoxicillin trihydrate/potassium clavulanate, and roxithromycin and the second was treated with amoxicillin trihydrate/potassium clavulanate and roxithromycin.

Mr. B-K presented to the emergency department on day 9 of chemotherapy cycle 6, with a 2-week history of suprapubic pain, painful urinary frequency, nocturia, weak stream, and dysuria and a 24-hour history of urge incontinence and sharp thoracolumbar paraspinal pain.

Mr. B-K was in severe pain with suprapubic tenderness on examination. Oxybutynin was administered without effect.

Medications on admission included fluconazole 200 mg, valacyclovir 500 mg, sulfamethoxazole/trimethoprim, allopurinol 300 mg, pantoprazole 40 mg, oxycodone 5 mg, sodium citrotartrate sachets, metoclopramide 10 mg, lorazepam 1 mg, and various aperients.

Mr. B-K was pancytopenic (Hb 99 g/L, WCC 1.0 × 10^9^/L, neutrophils 0.8 × 10^9^/L, lymphocytes 0.2 × 10^9^/L, and platelets 89 × 10^9^/L), and renal function remained normal throughout admission. Urine microscopy revealed mild haematuria and sterile pyuria (urinary WCC > 100 × 10^6^/L) and urine culture for bacteria and* Candida* was negative.

A urological consultation was sought; however, cystoscopy was not performed due to neutropenia. A presumptive diagnosis of overactive bladder was made, and the patient was discharged on solifenacin 5 mg po qd for symptomatic relief.

After discharge from hospital, the patient's symptoms were progressively more severe and distressing. Ongoing investigation included urethral swab for herpes simplex DNA and varicella DNA which were negative. Urine was negative for* Chlamydia trachomatis* and* Neisseria gonorrhoeae *nucleic acid. Serology excluded active CMV (IgM negative), EBV, HBV, HCV, HIV, leptospirosis, mycoplasma pneumonia, VZV, Q fever, and* Bartonella henselae*. No test for adenovirus was performed. Renal function was normal, and a renal tract ultrasound excluded renal and postrenal causes for his symptoms.

**Figure 1:**
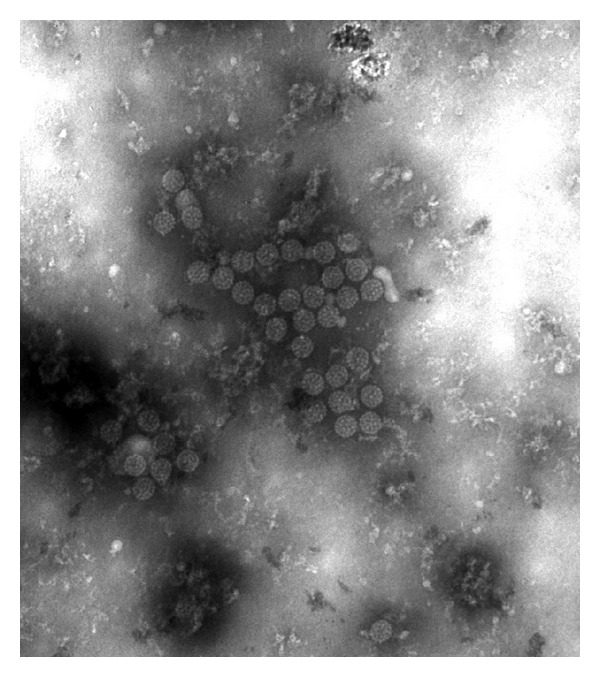
Viral inclusions seen on electron microscopy of urine epithelial cells.

Decoy cells were noted during urine cytological examination and electron microscopy was performed demonstrating intracellular viral inclusions suspicious for polyomavirus cystitis (Figure 1). Blood PCR for BK virus DNA was positive, with a viral load of 8316 copies/mL (PCR Taqman Probe) [1], consistent with a BK viraemia. Urine BK viral load is not routinely measured in our laboratory. Given the clinical symptoms, findings on urine electron microscopy, and time course, a presumptive diagnosis of BK virus disease was made. It is unknown whether this represented a primary infection or reactivation of latent virus.

In light of the limited evidence of efficacy of treatment with cidofovir, vidarabine, and leflunomide [2] for BK viral nephropathy and the potential renal toxicity of cidofovir, no specific antiviral therapy was initiated at time of diagnosis of BK viraemia. Cycle 7 of chemotherapy was withheld due to severe symptoms of cystitis and the patient showed significant clinical improvement. The risk benefit of continuing chemotherapy in terms of Hodgkin lymphoma control was considered at length and it was decided not to complete the last 2 cycles of chemotherapy due to the risk of ongoing BK virus infection with continued immunosuppressive therapy including cystitis and nephritis. The patient's symptoms settled over the next 2-3 weeks and he has been asymptomatic since.

Four months after BK viraemia was diagnosed, it was undetectable in the blood. The patient remains in radiological and clinical remission from Hodgkin lymphoma 28 months and 40 months posttreatment.

## 2. Discussion

BK virus is one of the polyomaviruses. Serological evidence of BK virus infection in 70–90% of asymptomatic healthy adults demonstrates its high prevalence in studied populations [2–4]. The primary route of transmission remains unclear, with both respiratory and oral routes proposed [2, 5]. Despite rarely presenting as a clinical problem, reactivation of latent BK infection in the renal tubular epithelial and urothelial cells can occur in the setting of cellular immunosuppression. A nonspecific inflammatory reaction is triggered by virus mediated cell lysis, and in immunocompetent hosts both cellular and humoural immunity is subsequently activated [4].

Symptomatic BK viremia is a relatively common finding following renal transplant and bone marrow transplant, in patients with HIV infection, and less common in heart and liver transplant recipients, with nephropathy or hemorrhagic cystitis the classic presentations [4, 6]. The relationship between immunosuppression and viral reactivation is complex [7], and it is not understood why symptomatic BK reactivation is reportedly only rarely a complication of chemotherapy alone. There are two case reports of symptomatic BK virus associated with Hodgkin lymphoma. The first is a case of viruria in a 15-year-old patient 2 weeks following chemotherapy. This patient had similar symptoms to our case; however, the patient did not have associated viremia [8]. A second case of renal failure with BK viruria in a 3-year-old child with Hodgkin lymphoma differed from our case in several ways. The child had the rare recessive disease cartilage-hair hypoplasia causing reduced numbers of T-lymphocytes, which may have independently explained the symptomatic BK virus infection. Furthermore, it is possible that this was a case of primary BK infection rather than reactivation of infection [9].

The question of whether BK virus is “peculiar to the kidney” in immunosuppression or a problem of immunosuppression generally, posed in the 1970s [10], has been superseded by the more clinically focused question: in whom does symptomatic, clinically significant disease occur [4]? Our case suggests that this question is still being answered.

Hirsch classifies three BK virus diagnostic states: the first is serological evidence of infection (BK virus infection), the second viral activity (BK virus replication), and the third symptomatic disease (BK virus disease) [2]. Leung et al. examined the correlation between BK viruria and subsequent hemorrhagic cystitis in a small study of patients receiving allogeneic hematopoietic stem cell transplants and concluded that viruria, although not directly causative, may be an important cofactor in the development of hemorrhagic cystitis [11]. This finding has since been confirmed by others [12–14] and has led to the practice of replication surveillance in transplant candidates.

In neutropenic patients, urinary tract infection cannot be excluded by negative urinary leukocytes and culture, cytology, and other tests are required. The presence of significant amounts of haematuria can be associated with severe thrombocytopenia, coagulopathy or viral infection (such as cytomegalovirus or adenovirus), or acute hemorrhagic cystitis (in patients receiving chemotherapeutics). Cyclophosphamide can cause hemorrhagic or nonhemorrhagic cystitis due to irritation of the bladder by the metabolite acrolein [6]. However, when related to cyclophosphamide use, it is dose dependent with onset within 48 hours of toxic dose initiation. Acrolein may cause asymptomatic damage to the bladder mucosa, setting up a susceptibility to viral infection in the immunosuppressed state that ensues [15]. Viral hemorrhagic cystitis is associated with immunosuppression, particularly of cellular immunity. It has been determined that BK virus specific T cells are undetectable in peripheral blood of immunosuppressed patients with polyomavirus associated nephropathy, however reappearing with immune reconstitution [5].

Diagnosis of BK related cystitis is difficult. Due to the ubiquitous nature of BK virus in the general population, serology is not helpful. Urine cytology may reveal decoy cells (enlarged nucleus with basophilic nuclear inclusions); however, it may be difficult to differentiate from malignancy, and it does not distinguish between the polyomaviruses. Urine polymerase chain reaction does not distinguish latent from active infection. Viral culture is not clinically useful due to speed of growth. Urine polymerase chain reaction (PCR) is a poor disease correlate as asymptomatic shedding is common. Plasma polymerase chain reaction is the standard for diagnosis of BK viraemia and correlates with nephropathy and inversely with immune status [16]. In a prospective study, Erard et al. reported that BK plasma viral loads >10^4^ copies/mL were predictive of hemorrhagic cystitis [14]. In the present case, the highest viral load was below this threshold; however, viral load may have peaked prior to diagnosis. The diagnosis of BK virus hemorrhagic cystitis is best made by urine viral load in the context of clinical symptoms.

Current treatment recommendation for BK viraemia depends on the organ or organs involved. There is little evidence supporting use of antivirals in polyomavirus associated nephropathy or hemorrhagic cystitis [5]. Studies reveal that traditional antivirals have no efficacy, and evidence for use of intravenous cidofovir has been difficult to extract due to the potential confounding effect of reducing immunosuppression. Recommended management of renal disease involves reduction, substitution, or discontinuation of immunosuppressive treatments, which was the strategy employed for our patient. Other authors have managed bladder involvement by irrigation and symptom control [5] or local bladder treatment with cidofovir with good effect; however, replication of these findings is needed [17].

## 3. Conclusion

This case identifies BK disease in a patient having aggressive chemotherapy for high risk Hodgkin lymphoma. Classically BK disease presents in posttransplant patients; however, with the introduction of aggressive and innovative therapies consideration must be given to atypical infections in symptomatic patients. BK disease warrants consideration as a differential in patients undergoing aggressive curative treatment regimens who develop symptomatic cystitis.
